# Production of Resistant Starch by Roasting Retrograded Starch with Glucose

**DOI:** 10.3390/molecules29122883

**Published:** 2024-06-18

**Authors:** Małgorzata Kapelko-Żeberska, Tomasz Zięba, Marta Meisel, Krzysztof Buksa, Artur Gryszkin

**Affiliations:** 1 The Faculty of Food Science, Wroclaw University of Environmental and Life Sciences, Chełmońnskiego 37/41, 51-630 Wrocław, Poland; tomasz.zieba@upwr.edu.pl (T.Z.); marta.meisel@upwr.edu.pl (M.M.); artur.gryszkin@upwr.edu.pl (A.G.); 2 Department of Carbohydrate Technology and Cereal Processing, University of Agriculture in Krakow, Balicka 122, 30-149 Krakow, Poland

**Keywords:** retrograded starch, glucose, roasting, resistant starch

## Abstract

Starch is a natural plant raw material applicable in many areas of industry. In practice, it is most often used in a modified form, i.e., after various treatments aimed at modifying its properties. Modifications of native starch enable producing resistant starch, which, as a prebiotic with confirmed health-promoting properties, has been increasingly used as a food additive. The present study aimed to determine the effect of roasting retrograded starch with the addition of anhydrous glucose at different temperatures (110, 130 or 150 °C) and different times (5 or 24 h) on the modified starch’s properties. The results of high-performance size-exclusion chromatography coupled with refractive index detector (HPSEC/RI) analysis and the changes observed in the solubility of starch roasted with glucose in DMSO, as well as in its other properties, confirm the changes in its molecular structure, including thermolytic degradation and the ongoing polymerization of starch with added glucose.

## 1. Introduction

Modified starch preparations are present in a wide array of food additives. Starch preparations modified via chemical and physical methods or using mechanical factors and their combinations [1,2] exhibit different properties compared to natural starches, which contribute to the beneficial and desired physicochemical properties of the food products they are used in. Such starches may serve as fat replacers and also strengthen fibers, bind water, and impart desired sensory and structure-forming traits [3]. The aforementioned modifications may also alter the patterns of digestion and absorption of the modified preparations featuring the properties of resistant starch (RS) [4], which is not completely digested in the small intestine of a healthy person and thus reaches the colon, where it is fermented by the local microflora [5] and therefore serves as a prebiotic. The various mechanisms of starch’s resistance to enzymatic digestion enable classifying it as one of five types of resistant starch (RS). And so, RS1 is a constituent of whole-meal products and escapes digestion by enzymes in the small intestine, remaining intact [6]. Granules of RS2 starch have a relatively compact structure, which significantly impairs their degradation by digestive enzymes. This is why this starch is called raw starch and is found, among other things, in raw potatoes or bananas [6]. In turn, RS3 is retrograded starch that is found in boiled and cooled potatoes and bread. Retrogradation occurs during the cold storage of starch paste and involves the re-association of amylose and amylopectin chains into ordered structures. After lowering the temperature, the colloidal starch solution formed during its pasting undergoes gelatinization. As a result, the solid starch phase forms a mesh structure that binds the aqueous phase in the meshes. During gel storage, the parallel double helices of starch chains are aggregated to form thermostable crystalline structures that exhibit resistance to the action of amylolytic enzymes [7,8]. RS4 starches represent a group of starches modified with physical or chemical methods or a combination thereof, which alter the structure of starch chains, thus limiting enzymes’ access to these chains. Both physical and chemical modifications, such as roasting, extrusion, acetylation and hydroxypropylation, increase starch’s resistance to amylases. Starch’s resistance to the action of amylolytic enzymes increases along with the increasing number of simultaneous chemical and physical modifications. In turn, RS5 is a type of resistant starch resulting from the formation of amylose–lipid complexes, which can be formed naturally or during food processing [9,10]. In the present study, starch was treated with physical agents (roasting at various temperatures), and this treatment was aided by glucose addition. Ample research works have demonstrated that exposure of starch to high temperatures triggers changes in its properties as well as in its digestion and absorption mechanisms in the human body [11,12,13]. Therefore, it would be interesting to investigate the use of glucose as a factor influencing the intensification of these changes.

Today, the majority of resistant starch preparations are produced through chemical modification, which is receiving increasingly negative feedback from consumers. In contrast, physical methods of starch modification have gained widespread acceptance in the population due to their low cost and safety of the final preparations. Physically modified starch can be safely used in the food, chemical and pharmaceutical industries. Physical modification of starch via roasting, drying, extrusion, hydrothermal treatment (HMT) or mechanical force leads to the disruption of the polymer structure, which ultimately modifies starch’s properties. Starch roasting and HMT are the most frequently deployed methods that modify its properties, such as its crystallinity, water absorption ability and pasting formation ability [11,14].

The present study aimed to determine the effect of roasting retrograded starch with the addition of anhydrous glucose at different temperatures (110, 130 or 150 °C) and for different durations (5 or 24 h) on the properties of the modified starch preparations.

## 2. Discussion of Results

### 2.1. Determination of Particle Size Distribution via Gel Chromatography

**Figure 1:**
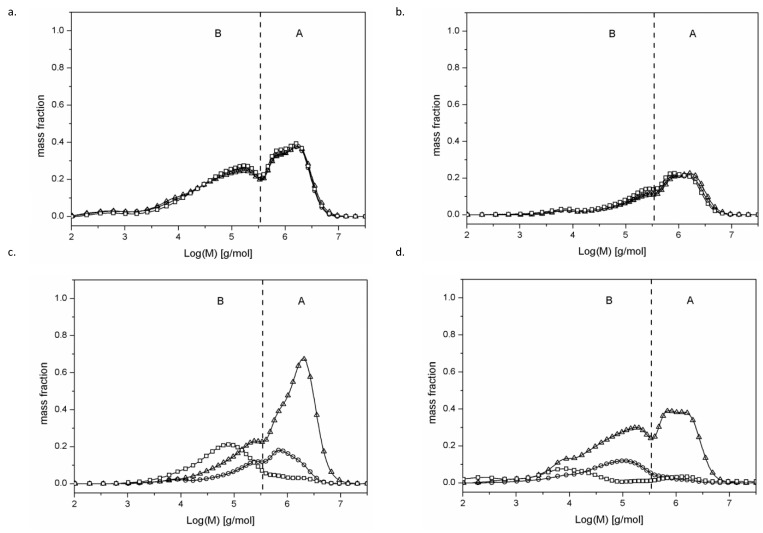
Molar mass distribution profiles of (**a**)—110/5 (-Δ-), 130/5 (-O-) and 150/5 (-□-) samples; (**b**)—110/24 (-Δ-), 130/24 (-O-) and 150/24 (-□-) samples; (**c**)—G110/5 (-Δ-), G130/5 (-O-) and G150/5 (-□-) samples; (**d**)—G110/24 (-Δ-), G130/24 (-O-) and G150/24 (-□-) samples. Starch fractions of high (A) and low (B) molar mass.

Starch is a substance of diverse molecular structures. Starch granules of various sizes and shapes are made up of branched amylopectin molecules with a molar mass of 10^7^–10^8^ g/mol, a much smaller straight-chain amylose with a molar mass of 10^5^–10^6^ g/mol and small amounts of protein, fat, water and mineral compounds physicochemically related to the carbohydrate fraction. The contents of amylose and amylopectin and other components of starch granules, and their spatial arrangement, which impart pseudocrystalline properties to starch, depend on the botanical origin of starch and significantly affect its functional properties [3,15,16]. Even minor exposure of starch to physical, chemical or biochemical factors affects its structure and, consequently, its physicochemical properties [17,18]. In the conducted experiment, retrograded potato starch was roasted with glucose, assuming that the long-term presence of a significant amount of this complete starch degradation product may affect the properties of roasted starch. Potato starch was used in this study due to its natural purity [19], whereas retrograded starch—due to its more porous developed structure [20] compared to that of native starch—ensures good contact of glucose with starch. During retrogradation, the straightened chains of water-dissolved amylose gradually strand into helices, which in turn merge into double helices reinforced with hydrogen bridges and additionally undergo dehydration (Figure S1). Aggregation of these helices occurs within a few hours of gel storage. Adjacent double helices form solid, thermostable, water-insoluble crystalline structures. Amylopectin also undergoes retrogradation, which is, however, a long-lasting process in this case. The crystalline products of amylopectin retrogradation are less thermally stable due to their branched structure and shorter chains (on average about 15 glucose residues), forming double helices [21]. As a result of the experiment, samples of retrograded starch roasted at various temperatures with and without the addition of glucose were obtained. However, no samples of glucose roasted at different temperatures were obtained. All these samples turned out to be completely soluble in the cold water used in the process of rinsing the prepared preparations. Table 1 presents the results of determinations of the molar mass distribution and values of the dispersion coefficient of retrograded starch roasted with and without glucose addition (Ð = Mw/Mn; Mw—weighted average molar mass and Mn—number average molar mass), which is a measure of the homogeneity of fractions constituting a given polymer [22]. Due to the relatively high values of this coefficient, reaching up to 13.9, the chromatogram was divided into two parts: part A for the high-molecular fraction and part B for the low-molecular fraction. The division line of these fractions is at about 500.000 g/mol (Figure 1). The molar mass of retrograded starch (Table 2) was higher than that of the retrograded starch that had been roasted and washed with water (Table 1). This observation is indicative of starch thermolysis which occurs during its roasting. The phenomenon of thermolysis, resulting in a reduced molar mass of starch, has been widely addressed in the scientific literature [23]. Interestingly, the analysis of molecular masses of starch roasted without glucose at various temperatures for 5 h and then rinsed with water showed no effect of the roasting temperature on the determined molecular masses compared to both the total mean molar mass (721–777 g × 10^3^/mol) and the mass of fraction A (1.363–1.454 g × 10^3^/mol) and fraction B (114–122 g × 10^3^/mol) granules. The results obtained in the present study are difficult to compare with the literature data addressing the temperature’s impact on the intensity of thermolysis [24], because they refer to starch that had only been roasted, and not roasted and washed with water. Roasting prolongation to 24 h caused an increase in the total mean molar mass (830–1.022 g × 10^3^/mol) and the mass of fraction B (152–155 g × 10^3^/mol), and similar values were noted for fraction A (1.287–1.463 g × 10^3^/mol). Probably, the roasting of starch also resulted in the cracking of starch chains, and the removal of low-molecular-mass water-soluble dextrins formed in the rinsing process, which could produce the apparent effect of no changes during the 5 h of roasting at different temperatures and an increase in the molar mass of starch during 24 h of roasting. It should be noted, however, that there were no major changes in the MW molar mass of fraction A granules during roasting, regardless of the duration and temperature. It can be speculated that low-molecular-mas amylose breaks down during starch thermolysis, and the amylopectin molecule is reduced by the cleavage of numerous outer low-molecular-mass starch chains. Amylopectin cracking inside the molecule can be excluded, as this would be reflected in a drastic molar mass decrease. Regardless of the roasting duration and temperature, the inner part of amylopectin turned out to be thermostable under the applied experimental conditions.

Roasting starch with the addition of glucose caused an unexpected change in its properties. Starch heated at 110 °C with glucose, compared to the glucose-free sample, had a different molar mass distribution. After 5 h of roasting, there was an increase in the MW of both starch fractions and in the total average molar mass. Thus, the thermolytic changes occurred both in fraction B (low-molecular-weight amylose—up to 500.000 g/mol) and fraction A (high-molecular-weight amylopectin—above 500.000 g/mol). The lower-molecular-weight carbohydrate structures cracked in each fraction. In the case of fraction A, the smallest particles (with a molecular weight slightly higher than 500.000 g/mol) underwent thermolysis and passed to fraction B, which in turn increased the MW value both in fraction A (where only particles larger than 500.000 g/mol remained) and fraction B. In turn, by reducing their molecular weight to slightly less than 500.000 g/mol (when they were perceived as large), these particles increased the MW value of this low-molecular-weight fraction.

Roasting extension to 24 h resulted in greater changes in the starch structure and, consequently, in lower molar masses of its molecules. When interpreting the effect of glucose on roasted starch, it is necessary to recall how the experiment was carried out. Retrograded starch served as the substrate in the experiment. Glucose present in the aqueous solution in large quantities compared to starch (1:1) was applied onto retrograded starch, and then the sample was conditioned for close to 24 h. This is a long enough time for water-soluble glucose to attach to retrograded starch molecules. Afterwards, the sample was dried, and glucose was crystallized locally due to solvent removal. Roasting starch with glucose at 130 or 150 °C resulted in its reduced solubility in DMSO (a solvent commonly used in SEC [25,26]) under the analytical conditions. This reduction in solubility indicates significant structural changes in the analyzed starch samples. These were not just thermolytic changes, as they improve the solubility of starch. It may be speculated that glucose molecules entered into reaction with starch molecules at higher temperatures because it has long been known that the formation of short-chain dextrins and glucose during long-term roasting of starch is followed by repolymerization reactions as a result of starch thermolysis [27,28]. These reactions result in the formation of branches at carbon atoms 2 and 3, which are not specific to starch [29]. In the experiment described in this manuscript, starch and glucose could undergo these changes from the very beginning of roasting. The outcomes of these transformations included, undoubtedly, a reduced starch solubility in DMSO, observed as early as 5 h after roasting at 130 or 150 °C, and its almost complete absence after 24 h of this process. Analogous changes were not observed in the case of the control samples (starch roasted without glucose addition). Hence, it may be concluded that glucose addition intensified these reactions. Starch chains have a spiral structure, where a single spiral is formed by about six glucose units. The secondary hydroxyl group (at the sixth carbon atom) is located on the outer side of the spiral, and the primary group (at the second and third carbon atoms) is located inside the spiral [30]. Thus, the attached individual glucose molecules will be located inside the starch spiral, physically forming “terminals” grafted within the starch helix. Assuming that there will be “terminals” in these physically twisted spiral structures, their physical untangling during dissolution is very difficult. Even a small number of these “terminals” that prevent the unfolding of one double spiral impairs the dissolution of a larger aggregated crystalline structure. This may also be the cause of the drastic decrease in the solubility of glucose-roasted starch in DMSO. Theoretically, there could also be reactions between the glucose molecules themselves, just like between starch and glucose. However, the products of these reactions turned out to be completely soluble in cold water (as evidenced by the failure to obtain roasted glucose samples), and were removed during the rinsing of the preparation and not detected in SEC analysis.

### 2.2. Swelling Power and Solubility in Water

**Figure 2:**
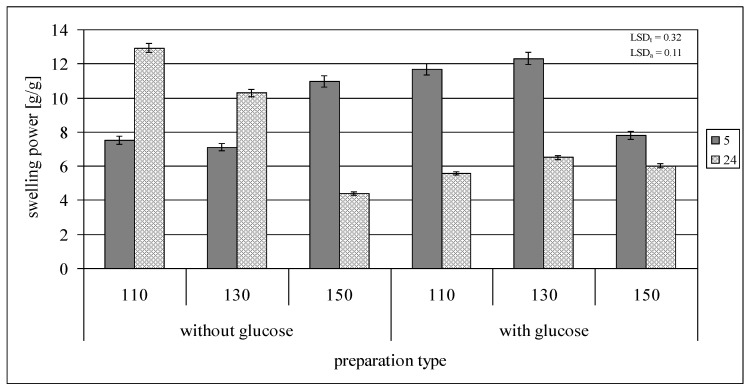
Swelling power of preparations of retrograded starches roasted at different times without or with glucose.

**Figure 3:**
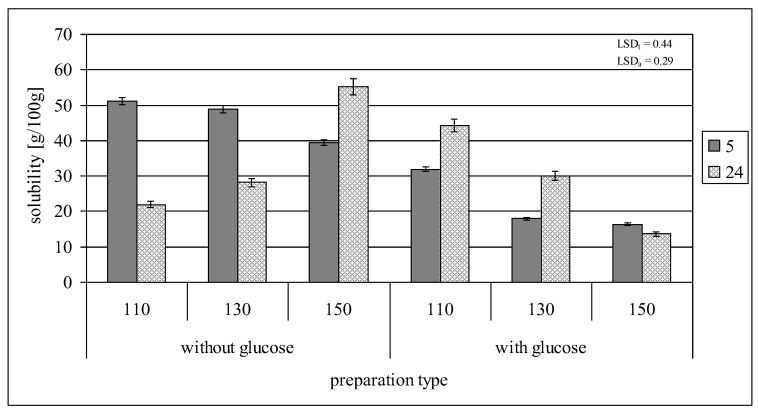
Solubility of preparations of retrograded starches roasted at different times without or with glucose.

Retrograded starch used in the experiment exhibited a 22.9 g/g swelling power and a 14.9 g/100 g solubility in water, determined at 80 °C. The five-hour roasting of starch at 110 or 130 °C, followed by its subsequent rinsing in cold water, resulted in a significant increase in its solubility (up to about 50 g/100 g) while reducing its swelling power (up to 7.1–7.5 g/g) at the temperature of determination (Figure 2 and Figure 3). The reasons for these changes should be found in the significantly reduced molar mass of starch under the influence of thermolysis. Similar correlations have been extensively described in the scientific literature [2,3,16,31]. Increasing the roasting temperature to 150 °C reduced starch’s solubility in water to ca. 40 g/100 g while increasing its swelling power to ca. 12 g/g. These changes were, however, not reflected in the reduction in the molar mass of starch and were most likely due to the structural changes of starch initiated under these conditions, i.e., the attachment of glucose units as a result of repolymerization of glucose molecules, which impaired the dissolution (unwinding) of double starch helices. The extension of the roasting time to 24 h at both 110 and 130 °C decreased starch’s solubility in water compared to the samples roasted for 5 h, but increased its swelling power; the observed changes intensified at a lower roasting temperature. The opposite trend was observed for starch roasted at 150 °C. It is worth noting that both thermolysis and glucose attachment occurred during starch roasting and that the intensity of these processes probably varied depending on the roasting conditions. The properties of starch roasted at a specific temperature and time were determined by the resultant effect of both processes. A second increase in solubility and a decrease in swelling power observed in the case of the starch preparation roasted at 150 °C for 24 h were probably due to the enhanced starch degradation, which was reflected in the lowest molar mass of this starch compared to the other starch samples roasted without glucose. The presence of glucose during starch roasting significantly affected the dynamics and trends in changes in both its solubility in water and swelling power. The five-hour roasting of starch with glucose at 110 °C increased its solubility (up to about 30 g/100 g) compared to the non-roasted retrograded starch, but this change was not as significant as in the case of starch roasted at this temperature without glucose. Increasing the roasting temperature to 130 or 150 °C caused a reduction in starch solubility by several percent, i.e., to a 2–3 times lower value, compared to the control samples. The changes in the swelling power of starch roasted for 5 h with glucose at different temperatures also differed from those observed for the control samples. The samples roasted at 110 or 130 °C were characterized by a higher swelling power and those roasted at 150 °C were characterized by a lower swelling power than the samples roasted without glucose. During twenty-four hours of starch roasting with glucose, its swelling power did not change significantly (it reached ca. 6 g/g), and the trends in changes in starch solubility with increasing roasting temperature were opposite to these observed for the control samples. Such a strong influence on the trends and magnitude of changes in the starch properties indicates that glucose addition during starch roasting not only affected the extent of thermolysis, but also modified starch’s molecular structure by embedding glucose molecules into the structure of its chains.

### 2.3. Differential Scanning Colorimetry

These structural changes of starch also had an impact on the characteristics of paste re-formation (“gelatinization”) by retrograded starch (Table 3). The non-roasted sample “gelatinized” at a lower temperature of 45.1–53.3 °C (Table 2) and in a narrower temperature range (8.2 °C) than the starch roasted without glucose addition. Out of the samples roasted without glucose, the highest “gelatinization” temperatures of about 50–67 °C were recorded for the samples roasted at 150 °C, regardless of the roasting time, with a significantly extended range of temperatures for this transition (17 °C). The enthalpy of gelatinization depended on the roasting conditions, and the highest values (approximately 8 J/g) were achieved, as in the case of pasting temperatures, by the samples roasted at the lowest tested temperature. Changes in the pasting characteristics mainly indicate the degrading effect of roasting on the crystalline structures of starch [16,32,33]. The addition of glucose affected the range and trends in changes in the characteristics of starch “gelatinization”, which was especially noticeable in the case of the samples roasted for 24 h. The increase in the roasting temperature caused a decrease in the gelatinization temperature (in contrast to the control samples) while reducing the enthalpy of starch gelatinization. The effect of glucose addition on starch during its roasting was the most pronounced when comparing the pasting characteristics of the samples roasted at a temperature of 150 °C for 24 h. The starch roasted without glucose was characterized by higher gelatinization temperatures (50.35–66.81 °C) and enthalpy (7.91 J/g) than the starch roasted with glucose addition (47.03–66.52 and 0.49 J/g, respectively). Presumably, structural changes in the starch impaired starch chain development in crystalline regions, which was reflected in a much lower transition heat.

### 2.4. Resistance to Amyloglucosidase

**Figure 4:**
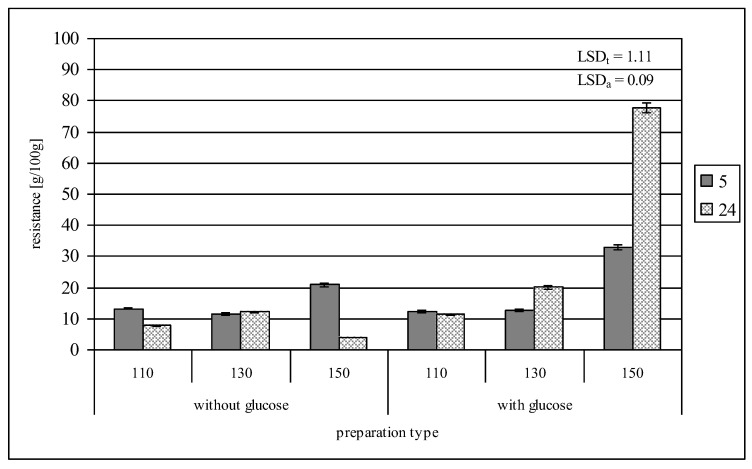
Resistance of preparations of retrograded starches roasted at different times without or with glucose to amyloglucosidase.

The analysis of resistance to amylolysis was conducted with amyloglucosidase of an enzymatic preparation called Dextrozyme used under industrial conditions for starch saccharification, which degrades both alfa 1,6 and alfa 1,4 glycosidic bonds in amylose and amylopectin chains. In our previous work [34], we performed respective analyses with an industrially used enzyme, as well as porcine alpha-amylase and glucoamylase, and showed differences in the results obtained. Retrograded potato starch showed 10 g/100 g resistance to amylolysis, which is consistent with the results presented by other authors [7,35,36]. Roasting this starch at 110 or 130 °C without glucose, regardless of the roasting time, caused no significant changes in its resistance (Figure 4). The insignificant changes observed in resistance might have been due to the impact of heat energy on the structure of retrograded starch, which might modify its primary resistance to RS3 starch. In addition, starch hydrolysis was followed by repolymerization. The final outcome of the observed changes is the result of these modifications, the intensity of which depended on roasting temperature and varied with time. The roasting starch for 5 h at 150 °C doubled starch’s resistance to amylases, whereas the 24 h roasting reduced it two-fold, which indicates the changes in the dynamics of modifications during roasting. The non-specific glucose–starch bonds that formed at carbon atoms 2 and 3 increased its resistance to enzymatic degradation, which is consistent with literature data [37]. The addition of glucose to starch during roasting significantly affected the changes in its resistance. During 5 h roasting even at 130 °C, the resistance of starch increased to 12.6 g/100 g, whereas after 24 h, it increased to 20.0 g/100 g. During 5 h roasting at 150 °C, as much as 1/3 of starch was not susceptible to enzymatic hydrolysis, whereas the 24 h roasting increased starch’s resistance to amylases to 77.8 g/100 g. Such a strong influence of glucose presence during starch roasting provides more evidence for the validity of our hypothesis, assuming that a change occurs in the spatial structure of starch chains under the applied experimental conditions. The “terminals” found inside the starch helix impair enzymes’ access to hydrolyzed bonds, increasing its resistance to amylolysis.

**Figure 5:**
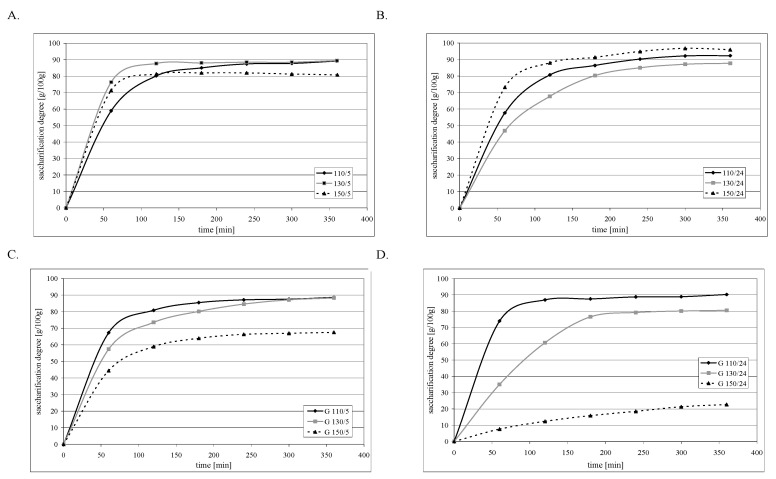
Dynamics of amyloglucosidase saccharification of preparations of retrograded starches at different temperatures: (**A**) 5 h without glucose; (**B**) 24 h without glucose; (**C**) 5 h with glucose; and (**D**) 24 h with glucose.

From a physiological standpoint, apart from the total resistance of starch to amylolysis, an important feature is the rate of starch digestion to glucose, which determines the so-called insulin response [38,39]. Figure 5A–D present the curves of starch saccharification dynamics. The starches roasted at 110 °C, regardless of their production method (roasting duration and glucose addition), were characterized by the highest saccharification rate. After about 2–3 h, all preparations reached their maximum saccharification. The rate of saccharification of the amylolysis-susceptible fraction of starch roasted at 150 °C was similar, even though this glucose-roasted starch exhibited significant resistance to the action of amylases. The strongest effect of starch preparation conditions was observed in the case of starch roasted at 130 °C, which was very quickly saccharified when roasted for 5 h without glucose. Extending the roasting duration to 24 h prolonged saccharification to 4–5 h and significantly decreased the rate of hydrolysis in the initial hours of this process. Presumably, at this temperature, low-molecular-mass carbohydrate chains were formed upon thermolysis, which afterward underwent repolymerization with starch, increasing its resistance to amylolysis. A similar effect could be observed in the sample roasted for 5 h at a temperature of 130 °C with glucose addition. In this case, repolymerization could proceed from the very beginning of roasting due to the presence of added glucose. In turn, the starch roasted for 24 h with glucose was characterized by an even release of glucose into the solution under the influence of amylolytic enzymes (the graph has a straight-line course within the first 3 h of hydrolysis), which distinguishes it from other starch preparations in which the greatest saccharification occurred in the first hour of hydrolysis. A similarly slow process of saccharification took place in the case of starch roasted with glucose at 150 °C, but here, the concentration of glucose in the solution was still very low due to its high total resistance (81 g/100 g).

Resistant starch is a prebiotic with confirmed health-promoting properties and has been increasingly used as a food additive [40,41,42]. The novel and at the same time easy method for resistant starch production proposed in the described experiment can be implemented under industrial conditions.

## 3. Materials and Methods

### 3.1. Materials

The experimental material was superior standard potato starch and anhydrous glucose produced in 2022 by the Przedsiębiorstwo Przemysłu Spożywczego PEPEES S.A. in Łomża (Łomża, Poland).

Dextrozyme DX 1.5X (DX), which contains glucoamylase (255 AGU/g) and pullulanase (510 NPUN/g), and Liquozyme Supra (LS), which is an alpha-amylase (135 KNU/g), were purchased from NOVONESIS (Lyngby, Denmark).

### 3.2. Production of Modified Starch Preparations

Aqueous suspensions, with a 7 g/100 g concentration, were prepared from native potato starch. The solutions were placed in a water bath at 94 °C for 6 h. The produced portions of pastes were left to cool for 24 h at a temperature of 20 °C. Subsequently, they were frozen at −18 °C for three days and thawed at 20 °C for two consecutive days. The excess water from precipitated starch with a spongy structure was drained off. The resulting retrograded starch was next dried at 35 °C in an air dryer for 48 h, ground, and sieved through a screen with a mesh size of 400 μm.

Afterwards, the produced retrograded starch was hydrated with a glucose solution at a concentration of 50 g/100 g or with water (reference sample) to achieve a starch-dry-matter-to-glucose ratio of 3:1. The sample was next conditioned at 20 °C for 24 h and dried successively in an air dryer at 35 °C for 48 h. Then, after cooling, the resulting mixture was divided into six portions and subjected to roasting at different times (5 or 24 h) and temperatures (110, 130 or 150 °C). The roasted preparations were cooled at 20 °C for 24 h, and then mixed with distilled water in a 3:1 ratio (water/preparation) and left for 5 h for sedimentation. This process was repeated 10 times. The preparations were dried in an air dryer at 35 °C for 48 h, cooled at 20 °C for 24 h, ground with a laboratory grinder, and sieved through a screen with a mesh size of 400 μm. Retrograded starch roasted without glucose, as well as glucose roasted without starch, subjected to the same treatments as the experimental samples, served as the control samples.

### 3.3. Determination of Particle Size Distributions via Gel Chromatography

The distribution of molar masses of the examined starches was evaluated by modified HPSEC/RI methods [43,44]. Prior to injection, 20 mg of the sample was mixed in 6 mL of DMSO at 70 °C for 24 h using a magnetic stirrer. Afterwards, the solutions were centrifuged at 2000× *g* for 5 min, and the supernatant was injected into the columns. The system consisted of a series of columns: OHpak SB-G (guard), OHpak SB-806, and OHpak SB-804 Shodex (Shimadzu Corporation, Kyoto, Japan). An aqueous solution of 100 mM NaNO_3_ was used as the eluent. The flow speed was 0.6 mL min^−1^, and the injection loop was 100 mL. The temperature of the columns was set at 60 °C. The molar mass distribution of starch was determined by refractive index (RI) detection. A calibration curve was plotted with pullulan standards (Shodex Standard, Macherey-Nagel, Düren, Germany) with known molecular masses (P-5, 10, 100, 400 and 800) and glucose. Each standard (10 mg) was dissolved in the eluent (4 mL) for 1 h and measured in the same way as the samples. The molar mass distribution was used to calculate the following molar parameters using Eurochrom (ver. 3.05, Knauer, Berlin, Germany) and Clarity (ver. 4.0.1.700, DataApex, Prague, Czech Republic) software: average molar mass (Mw, Mn) and dispersity (Ð = Mw/Mn).

The molar mass distribution profiles were divided into two fractions: fraction “A” of Mw > 500.000 g/mol and fraction “B” of Mw < 500.000 g/mol, and the molar parameters of each fraction were calculated.

### 3.4. Swelling Power and Solubility of Starch Preparations in Water at a Temperature of 80 °C

In brief, 200 mL of an aqueous suspension containing 1 g of starch preparations or modified starch preparations per 100 g of the solution was prepared in a round-bottom flask [44]. The flask was placed in a water bath and shaken at a temperature of 80 °C. It was kept under these conditions for 30 min until the flask reached the temperature of water bath temperature. Afterwards, the flask was cooled to a temperature of 20 °C, and water evaporated during heating was supplemented. Next, 50 g of the starch suspension was weighed into centrifuge tubes, which were then centrifuged in a Biofuge 28RS Heraeus Sepatech (Hanau, Germany) centrifuge at 14.500 rpm and 20 °C for 30 min. Next, the supernatant was decanted, and its dry matter content was determined using the air-dry method at a temperature of 105 °C. The precipitate left in the tubes was weighed [45].

### 3.5. Determination of the Characteristics of Phase Transitions of Starch Preparations with Differential Scanning Calorimetry (DSC)

The thermal pasting characteristics were determined using a Mettler Toledo DSC 822E scanning calorimeter and ME-5119872-type aluminum vessels (Columbus, OH, USA) with a capacity of 100 µL [34]. A 10 mg sample of the starch preparation was placed in a measuring vessel, to which redistilled water was added in a ratio of 3:1 (3 parts of water per 1 part of starch). The measuring vessels were conditioned at a temperature of 25 °C for 30 min, then heated to 100 °C at a heating speed of 4 °C/min. The obtained thermographs allowed determining the thermal characteristics of the tested samples, including the initial and final temperature of the phase transition and the mean specific heat of starch pasting [33].

### 3.6. Determination of the Resistance of Starch Preparations to Amyloglucosidase

A 0.72% starch suspension (38 g) was prepared in a conical flask, which was kept at the boiling point for 5 min [35]. After cooling the suspension, the volume of evaporated water was supplemented to the sample weight of 38 g, and then 34 mL of acetate buffer (pH = 4.35) was added to the flask. Next, the flask was placed in a water bath at 37 °C with active shaking, and 4 mL of a solution of an enzymatic preparation of Dextrozyme DX 1.5X (containing glucoamylase, α-amylase and pullulanase) was added (Dextrozyme DX 1.5X, NOVONESIS, Lyngby, Denmark). This amount of the enzyme was selected so as to ensure the complete saccharification of native starch after 2 h of hydrolysis. Every hour, 1 mL of the hydrolysate was put into a centrifuge vessel and centrifuged at 5.000 rpm for 5 min (with the final result being the value when the absorbance from three consecutive measurements remained unchanged). A total of 10 µL of the supernatant was collected from the centrifuged sample and transferred to the cuvette by adding 1 mL of a BIOSYSTEM reagent (Barcelona, Spain), then mixed and incubated at room temperature for 15 min. The absorbance was measured with a CECIL 2000 colorimeter (Villenave d’Ornon, France) at a wavelength of λ = 500 nm. Measurements were made against a blank sample, namely a reagent with buffer and water. The content of glucose was read out from the standard curve [34]. The resistance of the starch preparations was calculated from the following equation:

R = 100 − (x * 100)/0.396

where:

R—resistance of starch preparations [g/100 g].

x—content of glucose read out from the standard curve [mg].

0.396—the maximum amount of glucose formed from 0.36 g/100 g of the starch paste [mg].

### 3.7. Statistical Analysis

The experimental results were subjected to a statistical analysis using the Statistica 13.3 package (StatSoft, Cary, NC, USA).

The statistical computations (from at least three parallel replications) enabled determining the values of the least significant differences (LSDs) and standard deviations. For statistical evaluation, the results were subjected to a two-way analysis of variance at a significance level of 0.05. The values of the least significant difference (LSD) between the means were computed using Duncan’s test at a significance level of 0.05.

## 4. Conclusions

The addition of glucose to retrograded starch roasted for different periods (5 or 24 h) and at various temperatures (110–150 °C) significantly influenced the extent of and trends in the changes in its properties compared to the starch roasted without glucose addition. The differences in starch properties are indicative of both the ongoing thermolytic processes and the intense starch repolymerization with glucose caused by glucose addition. A significant increase in the resistance of starch roasted with glucose at 150 °C to amylolysis indicates the formation of bonds at the second and third carbon atoms that are non-specific to starch and hinder the enzymatic hydrolysis of starch chains.

## Figures and Tables

**Table 1:** Weighted average molar mass and degree of molar dispersity of preparations of retrograded starches roasted without or with glucose.

	110/5		130/5		150/5		110/24		130/24		150/24	
	Mw [g × 10^3^/mol]	Ð	Mw [g × 10^3^/mol]	Ð	Mw [g × 10^3^/mol]	Ð	Mw [g × 10^3^/mol]	Ð	Mw [g × 10^3^/mol]	Ð	Mw [g × 10^3^/mol]	Ð
whole	777	12.9	721	11.3	741	9.9	1022	8.5	926	8.8	830	9.3
A	1454	1.6	1363	1.5	1373	1.5	1463	1.6	1358	1.5	1287	1.5
B	114	9.1	117	8.7	122	6.5	155	3.6	155	5.3	152	6.4
	G110/5		(*) G130/5		(*) G150/5		G110/24		(-) G130/24		(-) G150/24	
	Mw [g × 10^3^/mol]	Ð	Mw [g × 10^3^/mol]	Ð	Mw [g × 10^3^/mol]	Ð	Mw [g × 10^3^/mol]	Ð	Mw [g × 10^3^/mol]	Ð	Mw [g × 10^3^/mol]	Ð
whole	1253	7.2	* 726	7.5	* 258	6.6	684	12.1	-	-	-	
A	1724	1.6	* 1155	1.5	* 1236	1.7	1330	1.5	-	-	-	
B	156	3.0	* 159	4.0	* 99	3.4	117	6.3	-	-	-	

(-) samples with negligible solubility, (*) partially soluble samples.

**Table 2:** Selected properties of retrograded starch.

Trait	Retrograded Starch
Molar mass MW [g × 10^3^/mol]	1643
Mw—fraction A [g × 10^3^/mol]	2415
Mw—fraction B [g × 10^3^/mol]	192
Swelling power [g/g]	22.87 ± 0.57
Solubility [g/100 g]	14.84 ± 0.37
Initial pasting temperature [°C]	45.12 ± 1.12
Final pasting temperature [°C]	53.34 ± 1.33
Phase transition heat [J/g]	6.04 ± 0.15
Resistance to amyloglucosidase [g/100 g]	10.04 ± 0.24

**Table 3:** Thermal properties of preparations of retrograded starches roasted without or with glucose, determined from DSC characteristics.

Type of Starch Preparation	Roasting Temperature [°C]	Initial Temperature of Gelatinization [°C]		LSD_T_	Final Temperature of Gelatinization [°C]		LSD_T_	Enthalpy of Gelatinization [J/g]		LSD_T_
		Roasting Time [h]			Roasting Time [h]			Roasting Time [h]		
		5	24		5	24		5	24	
without glucose	110	48.77 ^c^	45.85 ^d^	0.75	47.09 ^a^	64.48 ^b^	0.96	6.43 ^c^	6.01 ^c^	1.27
	130	50.45 ^a^	46.74 ^c^		64.64 ^b^	64.53 ^ab^		4.24 ^b^	6.68 ^a^	
	150	50.64 ^c^	50.18 ^a^		67.28 ^a^	66.70 ^ab^		7.80 ^a^	7.91 ^a^	
with glucose	110	50.21 ^b^	51.46 ^a^		65.89 ^b^	67.66 ^a^		8.04 ^a^	7.29 ^b^	
	130	49.15 ^b^	49.05 ^b^		63.86 ^a^	67.07 ^a^		5.90 ^a^	6.54 ^a^	
	150	46.94 ^ab^	47.06 ^a^		65.70 ^b^	66.50 ^b^		3.70 ^b^	0.48 ^c^	
LSD_A_		0.68			0.83			1.57		

Different letters mean homogeneous groups at *p* < 0.05.

## Data Availability

Data are contained within the article and Supplementary Materials.

## Supplementary Materials

The following supporting information can be downloaded at: https://www.mdpi.com/article/10.3390/molecules29122883/s1, Figure S1: Molar mass distribution profiles of native (-●-) and retrograded (-○-) starches.
