# Supplementary figures and images for: Production of Resistant Starch by Roasting Retrograded Starch with Glucose
Source: Molecules. 2024 Jun 18;29(12):2883. doi: 10.3390/molecules29122883 (PMC11207021; doi:10.3390/molecules29122883)

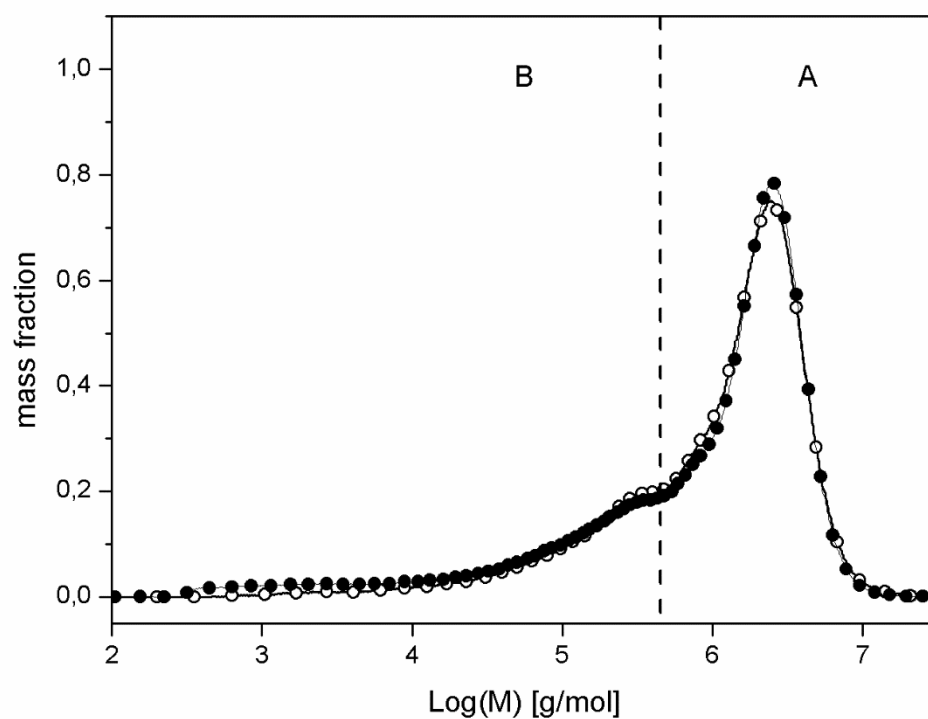

**Supplementary Figure S1.** Molar mass distribution profiles of native (●) and retrograded (○) starches.

Supplement: Supplementary file 1 [file molecules-29-02883-s001.zip › molecules-2993898-supplementary.pdf]
